# Steroid resistant, osimertinib induced hypereosinophilic syndrome (HES) treated with Benralizumab in a patient with metastatic adenocarcinoma of lung

**DOI:** 10.1016/j.rmcr.2025.102328

**Published:** 2025-11-19

**Authors:** M. Al Torkmani, H. Abid, Y. Al Kassar, R. Al Maashari, W. Gaba, S. Athar, M. Najib, M. Tufail

**Affiliations:** a Internal Medicine Department, Sheikh Khalifa Medical City, Abu Dhabi United Arab Emirates; b Pulmonology Department, Sheikh Khalifa Medical City, Abu Dhabi, United Arab Emirates

## 1 Introduction

Hypereosinophilic syndrome (HES) is rare and characterized by more than 1500 eosinophils/μL in peripheral blood. It can be primary due to clonal hematopoiesis, secondary to cytokines stimulating eosinophil production, or idiopathic. It may cause end-organ damage affecting various organs. With recent advances in targeted treatments for lung cancer, especially inhibitors of epidermal growth factor receptor (EGFR), prognosis has significantly improved [1]. These agents are generally safe, though hypereosinophilia associated with osimertinib has been reported [2]. Steroids have been the mainstay of treatment for such cases, and recently benralizumab, an anti-IL5R antibody, has been used for treatment of HES [3].

## 2 Case presentation

A 47-year-old woman was diagnosed with adenocarcinoma of the lung (T2N3M1b - Stage IV) in October 2022. Molecular testing from CT-guided biopsy confirmed an EGFR exon 19 deletion; therefore, osimertinib was started. The patient responded well to treatment. One year later (September 2023), she presented with shortness of breath and chest pain. She was found to be in type 1 respiratory failure, with arterial blood gas (ABG) showing pO_2_ of 60 mmHg on 10L O2. Laboratory investigations were unremarkable except for significant eosinophilia of 4920 cells/μL. A CT pulmonary angiography (CTPA) showed pulmonary emboli involving the segmental and subsegmental branches of the right lower lobe pulmonary artery. She was started on therapeutic anticoagulation.

**Fig. 1:**
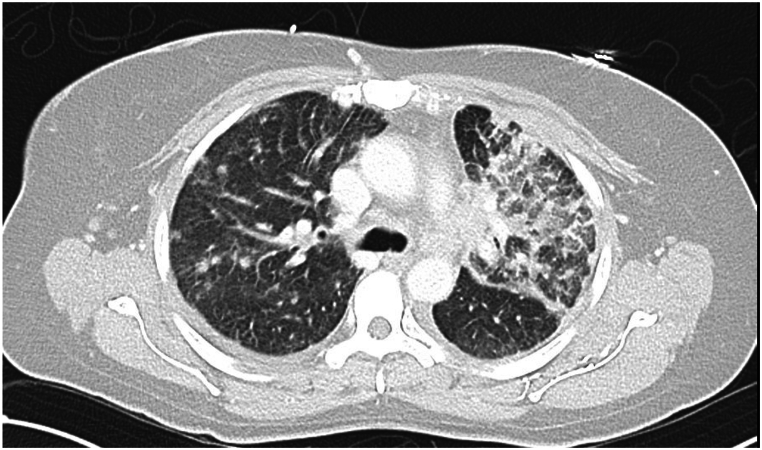
CT Chest showing bilateral lung infiltrates, predominantly in the left upper lobe.

On the second day of admission, bronchoscopy and bronchoalveolar lavage (BAL) were performed, which showed 21 % eosinophils, 17 % neutrophils, and 50 % lymphocytes. She was started on intravenous methylprednisolone 60 mg every 6 hours. She remained in respiratory failure, and a CT chest with contrast showed new bilateral infiltrates (Fig. 1). Her autoimmune screen and stool test for ova and parasites were negative, and serum Strongyloides antibodies were within the normal range. Therefore, osimertinib was considered the cause of hypereosinophilia after excluding all other possible causes, and hence it was discontinued.

**Fig. 2:**
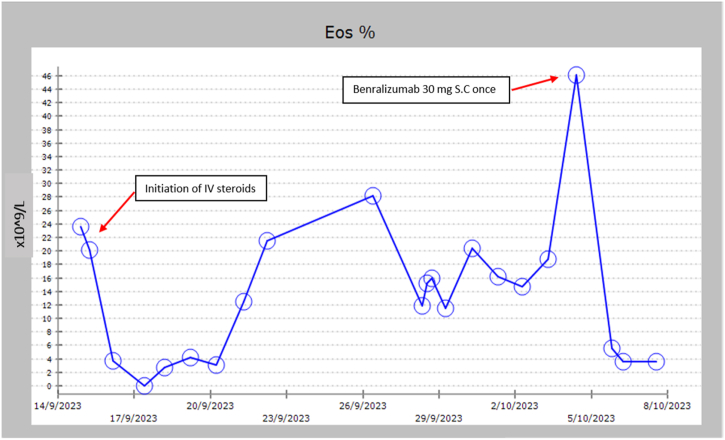
Absolute neutrophilic count at different points of treatment course.

The patient was continued on IV steroids, which initially suppressed the eosinophil count; however, upon switching to oral steroids, the eosinophil count rose again and respiratory symptoms worsened. One dose of Benralizumab 30 mg was administered subcutaneously, resulting in a significant drop in eosinophil count from 24 × 10^9^/L down to 2 × 10^9^/L within 2 days (Fig. 2, Table 1). The patient's respiratory status initially stabilized, and steroids were switched to an oral tapering regimen. She also developed left sided malignant pleural effusion which was drained with a chest tube. Unfortunately, the patient suffered a cardiac arrest four weeks into her admission in the hospital and passed away.

**Table 1:** Absolute eosinophil count and treatment at different points during the treatment course.

Date	09/15/2023	09/18/2023	09/21/2023	09/26/2023	09/30/2023	March 10, 2023	April 10, 2023	June 10, 2023
Absolute eosinophilic count	4.92 (x10^9/L)	0 (x10^9/L)	4.02 (x10^9/L)	10.10 (x10^9/L)	8.01 (x10^9/L)	8.30 (x10^9/L)	24.19 (x10^9/L)	1.15 (x10^9/L)
Treatment	Methylprednisolone 60mg q6hrly IV	Methylprednisolone 60mg q6hrly IV	Switched to PO Prednisolone 40mg daily	Methylprednisolone 60mg q6hrly IV (re-started)	Methylprednisolone 60mg q6hrly IV	Methylprednisolone 40mg q8hrly IV	Benralizumab 30mg and methylprednisolone 40mg q8hrly IV	Methylprednisolone 40mg q8hrly IV

## 3 Discussion

Hypereosinophilic syndrome (HES) is defined as a chronic increase in peripheral blood and/or tissue eosinophil levels, with an absolute eosinophil count ≥1.5 × 10^9^/L leading to end-organ damage [4]. HES is sub-classified into primary (clonal hematopoiesis), secondary (cytokine-mediated), or idiopathic when no cause is found. Secondary causes include infections (helminthic, fungal, viral), drugs, malignancies, and autoimmune diseases [5].

The main goal of treatment in HES is to reduce eosinophil counts and prevent end-organ damage. First-line therapy includes corticosteroids, usually initiated at 1 mg/kg/day of prednisone or up to 1 g methylprednisolone depending on severity [6]. Glucocorticoids act by suppressing eosinophil proliferation and activation [6]. However, their side-effect profile limits long-term use. Around 33 % of cases are steroid-resistant, where eosinophil counts are not persistently suppressed. Lack of response within 1–2 days is an indication to add another agent [7]. Interleukin-5 (IL-5) plays a key role in eosinophil differentiation and survival; thus, monoclonal antibodies targeting IL-5 can deplete eosinophils in blood and tissues [8].

One of the reported causes of HES is osimertinib, a main therapy for metastatic EGFR-positive non-small cell lung cancer. Osimertinib can cause pulmonary complications, including interstitial lung disease, pulmonary hemorrhage, and pneumonitis [9]. Eosinophilic pneumonia is characterized by eosinophil infiltration in lung tissue, confirmed by lung biopsy or BAL showing eosinophils >10 %, or peripheral eosinophilia >500 cells/μL, with radiographic pulmonary infiltrates [10]. Drug-induced eosinophilic pneumonia has been reported with amiodarone, daptomycin, sulfasalazine, and methotrexate [11]. Only three cases [9,12,13] of osimertinib-induced eosinophilic pneumonia have been described in the literature.

These cases, including ours, highlight the importance of early recognition of osimertinib-induced eosinophilic pneumonia. Patients typically present with cough, fever, dyspnea, and night sweats. CT findings are non-specific, often showing air-space consolidation and ground-glass opacities in a peripheral distribution [14]. As radiologic findings are non-specific, bronchoscopy remains the diagnostic standard. Steroids should be avoided before BAL, as they may hinder diagnosis. Discontinuation of the offending agent once drug-induced eosinophilic pneumonia is confirmed is essential to reduce morbidity and mortality [9].

## 4 Conclusion

Osimertinib-induced eosinophilia is a rare and poorly understood entity. In patients presenting with eosinophilic pneumonia and hypereosinophilic syndrome while on osimertinib, the drug should be considered a possible cause. The mainstay of treatment remains discontinuation of osimertinib and initiation of high-dose corticosteroids. In steroid-resistant cases, anti–IL-5 agents such as benralizumab can be considered.

## CRediT authorship contribution statement

**M. Al Torkmani:** Writing – review & editing, Supervision. **H. Abid:** Writing – review & editing, Writing – original draft. **Y. Al Kassar:** Writing – review & editing, Supervision. **R. Al Maashari:** Writing – review & editing, Supervision. **W. Gaba:** Writing – review & editing, Supervision. **S. Athar:** Writing – review & editing, Supervision. **M. Najib:** Writing – review & editing, Supervision. **M. Tufail:** Writing – review & editing, Writing – original draft, Supervision, Conceptualization.

## Declaration of competing interest

The authors declare that they have no known competing financial interests or personal relationships that could have appeared to influence the work reported in this paper.
